# Comparative optical characterization of renal cell carcinoma

**DOI:** 10.1007/s10103-026-04869-6

**Published:** 2026-04-21

**Authors:** Mehmet Bahadir Celik, Ibrahim Kucukkara

**Affiliations:** 1 Nanotechnology and Advanced Materials, Mersin University, Mersin, Turkey; 2 Department of Radiation Oncology, Mersin University, Mersin, Turkey; 3 Department of Physics, Mersin University, Mersin, Turkey

**Keywords:** Cancer, Diode laser, Renal cell carcinoma, Scattering coefficient, Absorption coefficient

## Abstract

Accurate characterization of the optical properties of renal cell carcinoma (RCC) tissue is essential for reliable modeling of light–tissue interactions in laser-based diagnostic and therapeutic applications. In this study, the absorption coefficient (µa), scattering coefficient (µs), anisotropy factor (g), and reduced scattering coefficient (µs′) of RCC tissues were systematically evaluated at wavelengths of 637, 785, and 850 nm under continuous-wave (CW) and 1000 ms pulsed diode laser illumination. RCC samples with thicknesses ranging from 0.8 to 2.0 mm were analyzed using a calibrated dual integrating sphere system to measure total reflectance, total transmittance, and unscattered transmittance under controlled and reproducible conditions. The optical parameters were reconstructed using the Inverse Adding–Doubling (IAD) method, and the effects of wavelength and laser modulation were statistically assessed using two-way ANOVA. The results demonstrated that wavelength significantly influenced µs and g, while µs’ exhibited a strong modulation-dependent response under pulsed illumination. In contrast, µa showed a monotonic spectral trend without reaching statistical significance. In addition, pulsed laser illumination resulted in a statistically significant increase in µs′ at 850 nm (*p* = 0.0286), indicating a modulation-dependent alteration in scattering behavior. Compared to reported values for healthy kidney tissue, RCC samples exhibited higher µa and µs′, along with lower µs and g values, reflecting microstructural changes associated with malignant transformation. This study represents the first multi-wavelength diode laser investigation integrating CW–pulsed modulation with IAD-based optical property reconstruction for RCC tissue. The findings provide experimentally validated optical parameters for improved light transport modeling and have direct implications for photodynamic therapy, photothermal therapy, and optical imaging applications involving renal malignancies.

## Introduction

Renal cell carcinoma (RCC) accounts for approximately 90% of all malignant renal tumors and remains a major clinical challenge due to its intrinsic resistance to radiotherapy and chemotherapy, high metastatic potential, and pronounced structural heterogeneity [1]. These characteristics necessitate the development of advanced diagnostic and therapeutic strategies capable of addressing both tumor complexity and treatment resistance. In this context, optical and laser-based technologies have emerged as promising tools for RCC diagnosis and treatment. Over the past two decades, techniques such as photothermal therapy (PTT), photodynamic therapy (PDT), optical biopsy, Raman spectroscopy, and diffuse reflectance spectroscopy have attracted increasing attention owing to their potential for minimally invasive tumor detection and targeted therapy. Other citiations edited Foster et al., [9] demonstrated the role of oxygen dynamics in photodynamic therapy. More recently, Gandhi and Lal [10], highlighted the clinical potential of near-infrared diode lasers in cancer imaging and treatment.

Accurate optical diagnosis and effective laser-based treatment critically depend on the precise quantification of tissue optical properties, including the absorption coefficient (µa), scattering coefficient (µs), anisotropy factor (g), and reduced scattering coefficient (µs′). Cheong et al., [7] provided one of the earliest comprehensive descriptions of optical properties in biological tissues. Later, Jacques [11], expanded this framework and established its relevance for modern biomedical optics applications. These parameters fundamentally govern photon transport within biological tissues, determining penetration depth, energy deposition, thermal confinement, and spatial light distribution. RCC tissues undergo substantial microstructural and biochemical alterations, such as cellular pleomorphism, abnormal angiogenesis, extracellular matrix remodeling, and the accumulation of lipid-rich clear cells. Li et al., [12] reported that microstructural alterations such as cellular pleomorphism and extracellular matrix remodeling significantly affect the optical signature of malignant renal tissue. Such pathological changes can markedly modify the optical signature of malignant tissue, emphasizing the necessity for accurate and tissue-specific optical characterization to improve light–tissue interaction modeling.

Despite the recognized importance of optical property determination, systematic studies on the optical characteristics of RCC tissue remain limited. Baran et al., [2] reported optical parameters of RCC for photodynamic therapy planning; however, their investigation did not incorporate multi-wavelength analysis or examine the influence of laser delivery mode. More recently, Botelho et al., [5] investigated the spectral optical behavior of chromophobe RCC and healthy kidney tissue, yet their study focused on broadband light sources and did not evaluate diode laser illumination or compare continuous-wave (CW) and pulsed irradiation conditions. This represents an important limitation, as pulsed laser exposure may transiently influence tissue optical responses through microthermal expansion, refractive index fluctuations, and modulation of scattering behavior, thereby affecting light transport and energy deposition. Chen et al., [6] demonstrated that pulsed laser irradiation can influence thermal and optical responses in biological tissues. Similarly, Tsai et al., [17] reported thermoelastic effects induced by pulsed laser exposure, which may alter scattering behavior.

To address these gaps, the present study performs a comprehensive multi-wavelength optical characterization of RCC tissue at 637, 785, and 850 nm using diode lasers operating in both continuous-wave and pulsed (1000 ms) modes. Tissue optical parameters were reconstructed using the Inverse Adding–Doubling (IAD) method. Prahl et al., [14] introduced the adding–doubling method for determining the optical properties of turbid media. Prahl [13], further developed and provided updated implementations of the Inverse Adding–Doubling (IAD) algorithm, which is a well-established and rigorously validated computational approach for determining the optical properties of turbid biological media from integrating sphere measurements. To the best of our knowledge, this is the first study to systematically evaluate the combined effects of wavelength and laser modulation on the optical behavior of RCC tissue. The findings provide experimentally derived optical parameters that are directly relevant for improving light transport modeling, optimizing laser dosimetry in PDT and PTT, and advancing the development of optical diagnostic techniques for renal malignancies.

## Materials and methods

### Tissue procurement and preparation

Human RCC tissue specimens were obtained from patients undergoing radical nephrectomy at affiliated medical centers, following approval by the institutional review board (IRB) and in accordance with established ethical guidelines for optical characterization of renal tissues. A total of three independent patient samples were included in this study. Tumor samples were collected within one hour post-excision to minimize dehydration and biochemical degradation, both of which are known to significantly alter tissue optical properties through water loss and chromophore oxidation. Macroscopically necrotic regions were carefully excluded to ensure representative measurements of viable tumor tissue, as necrosis introduces unpredictable absorption and scattering artifacts. The collected specimens were sectioned into uniform slices with thicknesses ranging from 0.8 to 2.0 mm using a precision microtome, thereby satisfying the assumptions of the Inverse Adding–Doubling (IAD) algorithm, which requires optically thin tissue layers to limit internal reflections and ensure accurate convergence. Prahl et al., [14] introduced the adding–doubling method for optical property reconstruction. Spinelli et al., [16] later applied similar approaches for bulk tissue optical characterization.

Each tissue slice was gently rinsed with isotonic saline to remove residual blood, which can disproportionately increase optical absorption in the visible spectral region due to hemoglobin content [3, 7]. The samples were subsequently positioned between two optically transparent microscope slides. To prevent compression-induced artifacts—known to artificially enhance scattering by modifying refractive index boundaries—500 μm spacers were placed around the tissue specimens to maintain a consistent and physiologically relevant thickness. Thickness uniformity was verified using precision spacers and optical inspection, and variations were maintained within ± 50 μm. Such small deviations are not expected to significantly affect the IAD-based optical property reconstruction. All optical measurements were completed within four hours of tissue extraction to preserve native optical behavior and minimize time-dependent optical drift that has been reported in ex vivo tissue studies. Repeated measurements performed during this time interval showed no statistically significant variation in optical parameters, confirming the temporal stability of the tissue optical properties under the experimental conditions. To minimize orientation-dependent effects, measurements were repeated at multiple positions across each tissue sample. No statistically significant variation was observed, indicating negligible influence of sample rotation on the reconstructed optical parameters.

### Laser illumination system

Three high-stability diode lasers operating at wavelengths of 637, 785, and 850 nm were employed for optical interrogation, corresponding to spectral regions commonly utilized in biomedical imaging and laser-based therapeutic applications due to their favorable tissue penetration characteristics and reduced absorption by endogenous chromophores. Each laser source was fiber-coupled and equipped with a 5 mm collimation output to ensure a spatially uniform beam profile at the sample surface, consistent with configurations reported in previous optical studies of renal and other soft tissues. Two distinct illumination modes were implemented. In the continuous-wave (CW) mode, laser radiation was delivered at constant optical power, representing standard conditions for steady-state photothermal monitoring and diagnostic reflectance spectroscopy. In the pulsed mode, square-wave irradiation was applied with a period of 1000 ms (1 Hz) and a 50% duty cycle while maintaining constant peak power, enabling the investigation of modulation-dependent optical responses. Pulsed laser excitation is known to generate transient thermal gradients and refractive index fluctuations within biological tissues, which may temporarily modify photon scattering behavior and effective light transport properties, thereby influencing the reconstructed optical parameters.

**Fig. 1:**
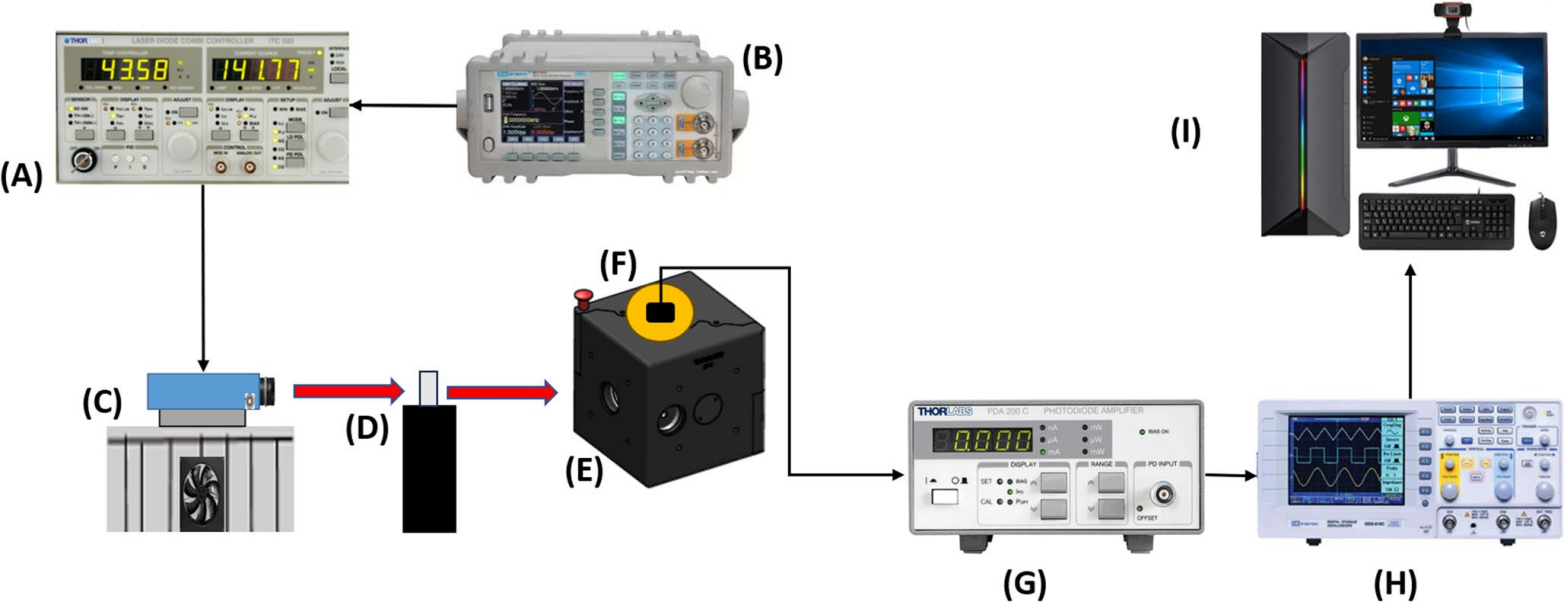
Schematic representation of the experimental setup used for optical measurements. The laser diode mounted on a cooling system **C** is driven by a Thorlabs ITC510 laser diode and TEC controller **A**, while temporal modulation is provided by an MFG-3010 function generator **B**. The tissue sample is positioned in the sample holder located at the integrating sphere input port **D**. The laser beam is directed into a Ø50 mm integrating sphere (Thorlabs 2P4) **E**. The optical signal is detected by a silicon photodiode (SM05PD2A) mounted at the detector port **F** and amplified using a PDA200C photodiode amplifier **G**. The amplified signal is monitored using an oscilloscope **H** and recorded via a PC-based data acquisition system **I**

Figure 1 has been removed, and all subsequent figures have been renumbered accordingly.

Diode lasers were selected due to their high stability, narrow linewidth, compact structure, and established role in minimally invasive phototherapies including PDT and PTT. Output power was calibrated using a thermal power meter following standardized laser safety and dosimetry protocols and maintained below 150 mW/cm² to avoid thermal denaturation or irreversible structural changes in tissue microarchitecture, consistent with prior thermal modeling studies. This power density was selected to ensure non-destructive optical measurements and to avoid thermal damage or structural alterations in the tissue, consistent with previously reported safe irradiation conditions in biological tissue optics [15]. The polarization state of the incident laser beam was not explicitly controlled. However, diode lasers are typically weakly polarized, and in highly scattering biological tissues such as RCC, polarization is rapidly randomized after multiple scattering events. Therefore, polarization effects are expected to have negligible influence on the reconstructed bulk optical parameters.

### Integrating sphere optical measurement system

A dual integrating sphere configuration was employed to measure angularly integrated reflectance and transmittance, following standardized methodologies widely adopted in optical tissue characterization studies [14, 16]. The experimental setup consisted of two integrating spheres, each with a diameter of 150 mm, configured for diffuse reflectance and diffuse transmittance measurements, respectively. The internal surfaces of both spheres were coated with barium sulfate, providing high diffuse reflectivity (ρ ≈ 0.98) and near-Lambertian scattering behavior with excellent optical stability across the visible and near-infrared spectral regions [4, 18].

High-sensitivity silicon photodetectors were mounted at standardized detection ports to ensure consistent angular integration of scattered radiation in accordance with established integrating sphere protocols [13, 19]. Using this configuration, three fundamental optical parameters were experimentally determined. Total reflectance (Rₜ), encompassing both specular and diffuse backscattering components, was measured to characterize superficial and bulk scattering behavior [11]. Total transmittance (Tₜ), representing the sum of all forward-scattered photons, was acquired to provide information on internal tissue scattering and absorption properties [7]. Unscattered transmittance (T_u_) was measured by isolating ballistic photons through narrow-aperture detection, a critical parameter for accurate Inverse Adding–Doubling (IAD) reconstruction and for identifying low-scattering transport regimes [16, 20].

Environmental conditions were carefully controlled throughout all measurements to maintain stable temperature and humidity, as tissue optical properties are highly sensitive to dehydration, thermal fluctuations, and refractive index variations [3, 8]. These precautions ensured measurement reproducibility and minimized temporal drift in reflectance and transmittance signals.

### Experimental system

The experimental setup used for optical measurements is schematically illustrated in Fig. 1. The laser diode mounted on a temperature-controlled cooling system (C) was driven by a Thorlabs ITC510 laser diode and TEC controller (A), while temporal modulation of the laser output was provided by an MFG-3010 function generator (B). The biological tissue sample was positioned in a dedicated sample holder located at the input port of a Ø50 mm integrating sphere (Thorlabs 2P4) (D, E). The incident laser beam entered the integrating sphere after interacting with the sample, enabling the collection of spatially integrated optical signals. The detected signal was acquired using a silicon photodiode (SM05PD2A) mounted at the detector port of the integrating sphere (F) and subsequently amplified by a PDA200C photodiode amplifier (G). The amplified signal was monitored using an oscilloscope (H) and recorded through a PC-based data acquisition system (I) for further analysis.

### Inverse adding–doubling (IAD) algorithm

The Inverse Adding–Doubling (IAD) algorithm developed by Scott Prahl was employed for optical property reconstruction, as it represents one of the most extensively validated and widely accepted computational approaches for extracting tissue optical parameters from integrating sphere measurements [13, 14]. In contrast to diffusion-based models, which rely on the assumption µs ≫ µa and are therefore limited to highly scattering media, the IAD method enables accurate reconstruction over a broad range of µa/µs ratios without invoking simplifying approximations [11, 18]. This flexibility renders IAD particularly well suited for the optical characterization of biologically heterogeneous tissues, including malignant tumors such as renal cell carcinoma.

The input parameters for the IAD model included experimentally measured total reflectance (Rₜ), total transmittance (Tₜ), and unscattered transmittance (T_u_), which collectively provide the angularly averaged boundary information required for inverse optical property reconstruction [16]. Additional inputs comprised the physical thickness of each tissue sample and the refractive indices of the tissue (n_tissue = 1.37) and glass slides (n_slide = 1.50), which are necessary for accurate modeling of Fresnel reflections at tissue–air and tissue–substrate interfaces [7]. Sphere wall reflectivity coefficients were also incorporated to correct for internal losses within the integrating spheres and ensure proper energy balance during reconstruction [4].

The IAD algorithm yielded the absorption coefficient (µa), scattering coefficient (µs), anisotropy factor (g), and reduced scattering coefficient (µs′ = µs(1 − g), which are the fundamental optical parameters governing photon transport in turbid biological tissues. Jacques [11], described the fundamental role of optical parameters in light–tissue interaction. Wang and Wu [19], further detailed their importance in biomedical imaging and modeling. Convergence was achieved when the relative difference between forward-calculated and experimentally measured reflectance and transmittance values was less than 0.1%, a criterion commonly adopted in IAD implementations to ensure high reconstruction accuracy for biological tissue measurements [13, 16].

### Statistical analysis

Statistical analysis was performed using two-way analysis of variance (ANOVA) to evaluate the independent and interactive effects of wavelength and laser modulation on the reconstructed optical properties. The first independent factor was wavelength (637, 785, and 850 nm), which is known to significantly influence tissue absorption and scattering through chromophore-specific absorption spectra and wavelength-dependent refractive index contrast [7, 11]. The second factor was laser modulation mode (continuous-wave versus pulsed), as temporal delivery conditions can induce transient thermal gradients and refractive index variations that affect light–tissue interactions [6, 17]. The dependent variables included the primary optical parameters reconstructed by the IAD algorithm, namely the absorption coefficient (µa), scattering coefficient (µs), anisotropy factor (g), and reduced scattering coefficient (µs′), each of which plays a distinct role in governing photon transport in biological tissues [18, 19].

A significance threshold of *p* < 0.05 was adopted, consistent with established statistical practices in biomedical optics research [8]. Where the overall ANOVA revealed statistically significant effects, post hoc pairwise comparisons were conducted using Tukey’s honestly significant difference (HSD) test. Particular emphasis was placed on µs′, as the reduced scattering coefficient is highly sensitive to tissue microstructural organization, including nuclear morphology, mitochondrial density, and extracellular matrix heterogeneity, and is therefore more responsive to modulation-dependent factors than µa or µs [11, 12]. Modulation-induced variations in µs′ have also been reported in thermoelastic and time-resolved optical studies, underscoring its relevance for characterizing dynamic light–tissue interactions [6, 17].

Post hoc statistical power analysis was conducted based on the observed effect sizes to assess the sensitivity of the two-way ANOVA. Statistical power exceeded 0.80 for µa, µs, and g, indicating adequate sensitivity to detect wavelength-dependent differences. For µs′, larger effect sizes were observed, and the corresponding statistical power exceeded 0.90, supporting the robustness of the modulation-dependent findings. Although the sample size was moderate, the combination of strong effect sizes and low within-group variance supports the reliability and interpretability of the statistical conclusions.

## Results

A total of 20 optical data sets were acquired for each condition, distributed across three independent RCC patient samples, with repeated measurements performed on each sample to ensure statistical reliability. All measurements exhibited excellent convergence of the Inverse Adding–Doubling (IAD) reconstruction, with reconstruction errors below 0.1%, confirming the reliability and numerical stability of the extracted optical parameters.

### Optical properties of RCC tissue at 637 nm

At a wavelength of 637 nm, 20 independent measurements were performed for each illumination mode (continuous-wave and pulsed), distributed across three independent RCC patient samples. The experimentally measured total reflectance (Rₜ), total transmittance (Tₜ), and unscattered transmittance (T_u_) values were processed using the Inverse Adding–Doubling (IAD) algorithm to reconstruct the absorption coefficient (µa), scattering coefficient (µs), anisotropy factor (g), and reduced scattering coefficient (µs′). These parameters were subsequently analyzed to evaluate the influence of laser modulation on the optical behavior of RCC tissue at this wavelength. The optical properties of RCC tissue at 637 nm under both continuous-wave and pulsed illumination are summarized in Table 1.

SD added CW and Pulse combined.

**Table 1:** Optical properties of RCC tissue at 637 nm under continuous-wave and pulsed illumination

Parameter	Symbol	CW (Mean ± SD)	Pulsed (Mean ± SD)
Absorption	µa	0.315 ± 0.015	0.325 ± 0.017
Scattering	µs	3.85 ± 0.12	3.80 ± 0.14
Anisotropy	g	0.34 ± 0.02	0.35 ± 0.02
Reduced Scattering	µs′	2.42 ± 0.10	2.44 ± 0.11

Under continuous-wave (CW) illumination at 637 nm, RCC tissue exhibited moderate absorption and comparatively low scattering behavior, consistent with the structurally heterogeneous nature of malignant renal tissue. The observed anisotropy factor (g = 0.34) indicates that photon scattering is substantially less forward-directed than typically reported for healthy kidney tissue, suggesting increased angular randomization associated with tumor-related microarchitectural disruption.

Under pulsed illumination at 637 nm, the reconstructed optical parameters were comparable to those obtained under continuous-wave conditions, with only minor increases observed in the absorption coefficient (µa) and reduced scattering coefficient (µs′). These small deviations are consistent with transient thermoelastic perturbations expected under millisecond-scale laser pulses; however, no statistically significant differences were detected between continuous-wave and pulsed modes at this wavelength (*p* = 0.48).

### Optical properties of RCC tissue at 785 nm

At 785 nm, the absorption coefficient decreased relative to 637 nm, primarily due to the reduced contribution of hemoglobin absorption in the near-infrared spectral region, while a slight increase in scattering-related parameters was observed. This wavelength is located near the lower boundary of the first near-infrared optical window, which facilitates deeper photon penetration into biological tissues and alters the balance between absorption and scattering in RCC tissue. The reconstructed optical properties of RCC tissue at 785 nm under both continuous-wave and pulsed illumination are summarized in Table 2.

**Table 2:** Optical properties of RCC tissue at 785 nm under continuous-wave and pulsed illumination

Parameter	Symbol	CW (Mean ± SD)	Pulsed (Mean ± SD)
Absorption coefficient	µa	0.229 ± 0.013	0.203 ± 0.015
Scattering coefficient	µs	4.06 ± 0.15	4.09 ± 0.17
Anisotropy factor	g	0.34 ± 0.03	0.26 ± 0.03
Reduced scattering coefficient	µs′	2.55 ± 0.12	2.93 ± 0.14

At 785 nm, pulsed illumination produced an increase in the reduced scattering coefficient (µs′) relative to continuous-wave conditions; however, this difference did not reach statistical significance when evaluated within the two-way ANOVA framework (*p* > 0.05). The accompanying decrease in the anisotropy factor (g = 0.26) indicates a shift toward more isotropic scattering under pulsed excitation. This behavior may be attributed to transient thermo-optic effects, such as short-lived refractive index gradients or microstructural swelling induced by rapid thermal modulation.

### Optical properties of RCC tissue at 850 nm

The optical parameters obtained at 850 nm under both continuous-wave and pulsed irradiation are summarized in Table 3.

**Table 3:** Optical properties of RCC tissue at 850 nm under continuous-wave and pulsed illumination

Parameter	Symbol	CW (Mean ± SD)	Pulsed (Mean ± SD)
Absorption coefficient	µa	0.261 ± 0.014	0.202 ± 0.016
Scattering coefficient	µs	4.03 ± 0.13	4.09 ± 0.16
Anisotropy factor	g	0.45 ± 0.03	0.25 ± 0.03
Reduced scattering coefficient	µs′	2.11 ± 0.11	2.93 ± 0.15

At 850 nm, pulsed illumination resulted in a pronounced increase in the reduced scattering coefficient (µs′ = 2.93 mm⁻¹), accompanied by a substantial reduction in the anisotropy factor (g = 0.25). This combination indicates a shift toward more isotropic scattering under pulsed excitation. Such changes may be attributed to transient thermo-optic effects induced by millisecond-scale laser modulation, including short-lived alterations in microstructural refractive index profiles that influence effective photon scattering pathways.

In contrast, under continuous-wave illumination at 850 nm, RCC tissue exhibited the lowest absorption among the investigated wavelengths, consistent with minimal hemoglobin absorption in the near-infrared spectral region. The higher anisotropy factor observed at this wavelength (g = 0.45) indicates more forward-directed scattering compared with 637 and 785 nm, reflecting the reduced refractive index contrast and increased photon penetration depth characteristic of near-infrared light propagation in biological tissues.

### Absorption coefficient (µa)

**Fig. 2:**
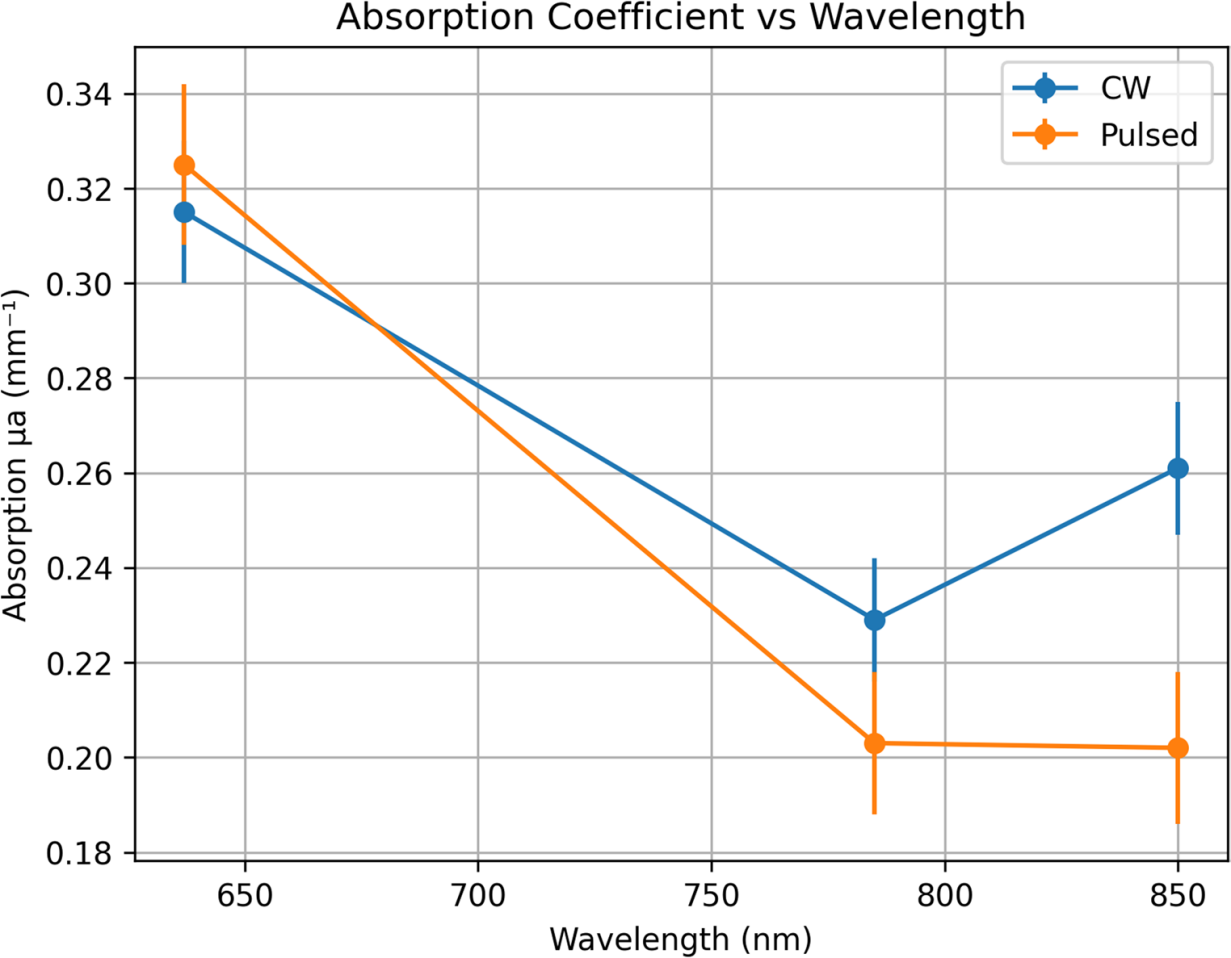
Absorption coefficient µa vs. wavelength (CW vs. Pulsed). Data are presented as mean ± SD (*n* = 20 measurements distributed across three independent samples)

The absorption coefficient (µa) exhibited a wavelength-dependent trend across the investigated spectral range; however, this variation did not reach statistical significance in the Two-Way ANOVA analysis (*p* > 0.05). At 637 nm, µa was measured as 0.315 mm⁻¹ under continuous-wave illumination and 0.325 mm⁻¹ under pulsed illumination. At 785 nm, µa decreased to 0.229 mm⁻¹ (CW) and 0.203 mm⁻¹ (pulsed). The lowest absorption values were observed at 850 nm, with µa equal to 0.261 mm⁻¹ for continuous-wave mode and 0.202 mm⁻¹ for pulsed mode. These results are consistent with reduced hemoglobin absorption in the near-infrared region and confirm that laser modulation does not significantly affect bulk chromophore absorption in RCC tissue. The wavelength-dependent variation of the absorption coefficient under continuous-wave and pulsed illumination is shown in Fig. 2.

### Scattering coefficient (µs)

The scattering coefficient (µs) exhibited a clear dependence on wavelength, while no statistically significant effect of laser irradiation mode was observed (*p* > 0.05). At 637 nm, µs was 3.85 mm⁻¹ under continuous-wave illumination and 3.80 mm⁻¹ under pulsed illumination. At 785 nm, µs increased to 4.06 mm⁻¹ (CW) and 4.09 mm⁻¹ (pulsed). At 850 nm, µs values were comparable between modes, measuring 4.03 mm⁻¹ for continuous-wave illumination and 4.09 mm⁻¹ for pulsed illumination.

**Fig. 3:**
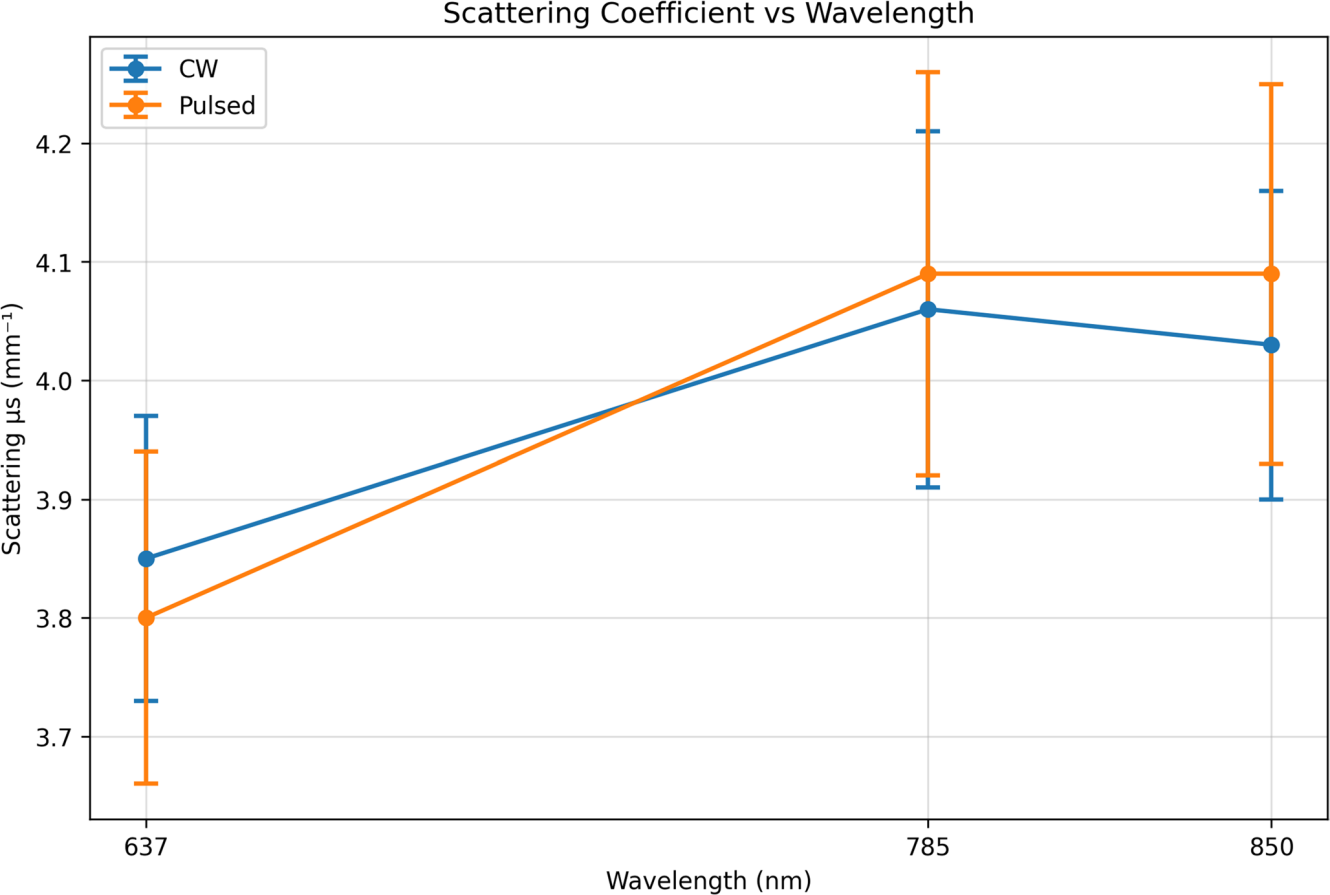
Scattering coefficient µs vs. wavelength (CW vs. Pulsed). Data are presented as mean ± SD (*n* = 20 measurements distributed across three independent samples)

These results indicate that the bulk scattering magnitude in RCC tissue is primarily governed by wavelength-dependent structural properties rather than by the temporal modulation of laser delivery. The absence of a statistically significant modulation effect suggests that millisecond-scale pulsed irradiation does not substantially alter the dominant scattering centers within the tissue, and that modulation-dependent effects are more likely to influence scattering directionality rather than its overall magnitude. The wavelength-dependent behavior of µs under both illumination modes is illustrated in Fig. 3.

### Anisotropy factor (g)

The anisotropy factor (g) exhibited a strong dependence on wavelength but was not significantly influenced by laser irradiation mode (*p* > 0.05). At 637 nm, g was measured as 0.34 under continuous-wave illumination and 0.35 under pulsed illumination, indicating relatively isotropic scattering. At 785 nm, g decreased to 0.34 (CW) and 0.26 (pulsed), suggesting a shift toward more isotropic scattering under pulsed conditions. At 850 nm, g increased to 0.45 under continuous-wave illumination, while pulsed irradiation resulted in a reduced anisotropy value of 0.25.

**Fig. 4:**
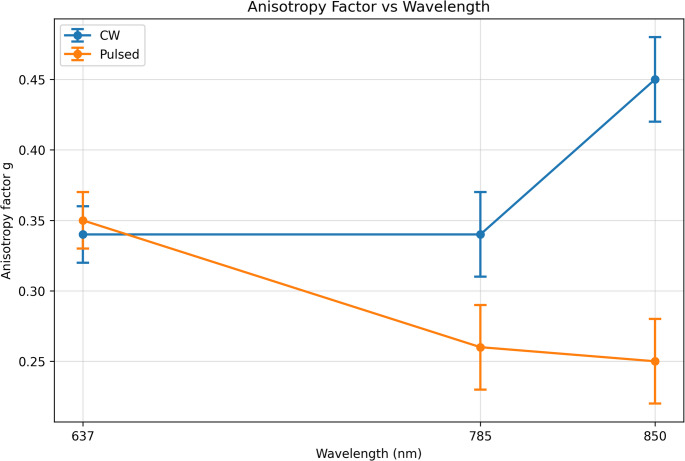
Anisotropy factor vs. wavelength (CW vs. Pulsed). Data are presented as mean ± SD (*n* = 20 measurements distributed across three independent samples)

This wavelength-dependent increase in g under continuous-wave conditions reflects enhanced forward-directed scattering at longer wavelengths, consistent with Mie scattering behavior in heterogeneous tumor tissue. According to Mie theory, larger intracellular structures—such as nuclei, mitochondria, and lipid vesicles—contribute more significantly to scattering at longer wavelengths, leading to increased anisotropy and greater photon penetration depth. These findings suggest that wavelength plays a dominant role in determining scattering directionality, while temporal modulation primarily affects secondary scattering characteristics. The wavelength-dependent behavior of the anisotropy factor under continuous-wave and pulsed irradiation is illustrated in Fig. 4.

### Reduced scattering coefficient (µs′)

Among all reconstructed optical parameters, the reduced scattering coefficient (µs′) exhibited the highest sensitivity to laser irradiation mode. At 637 nm, µs′ values were comparable under continuous-wave and pulsed illumination (2.42 and 2.44 mm⁻¹, respectively), indicating minimal modulation dependence at this wavelength. At 785 nm, µs′ increased from 2.55 mm⁻¹ under continuous-wave conditions to 2.93 mm⁻¹ under pulsed illumination. A more pronounced modulation-dependent effect was observed at 850 nm, where µs′ decreased to 2.11 mm⁻¹ under continuous-wave illumination but increased markedly to 2.93 mm⁻¹ under pulsed conditions. This increase at 850 nm was statistically significant (*p* = 0.0286), highlighting a strong interaction between wavelength and laser irradiation mode.

Although the increase in µs′ under pulsed illumination does not represent a strict two-fold change, the relative enhancement (~ 35–40%) is substantial in the context of biological tissue optics and exceeds the variability observed at shorter wavelengths. These findings indicate that µs′ is particularly sensitive to the temporal characteristics of laser delivery, especially in the near-infrared spectral region. While modulation-dependent changes in scattering-related parameters have been suggested in thermo-optic and time-resolved optical studies, such a pronounced µs′ enhancement under millisecond-scale pulsed diode laser illumination has not been widely reported for renal tissue.

This behavior may be attributed to transient refractive index fluctuations and short-lived micro-expansion of subcellular structures induced by pulsed thermal loading. Such effects can increase angular randomization of scattered photons without significantly altering bulk scattering magnitude or absorption, thereby selectively enhancing the reduced scattering coefficient. This selective sensitivity explains why µs′—rather than µs or µa—emerges as the most responsive parameter for probing dynamic light–tissue interactions in RCC.

**Fig. 5:**
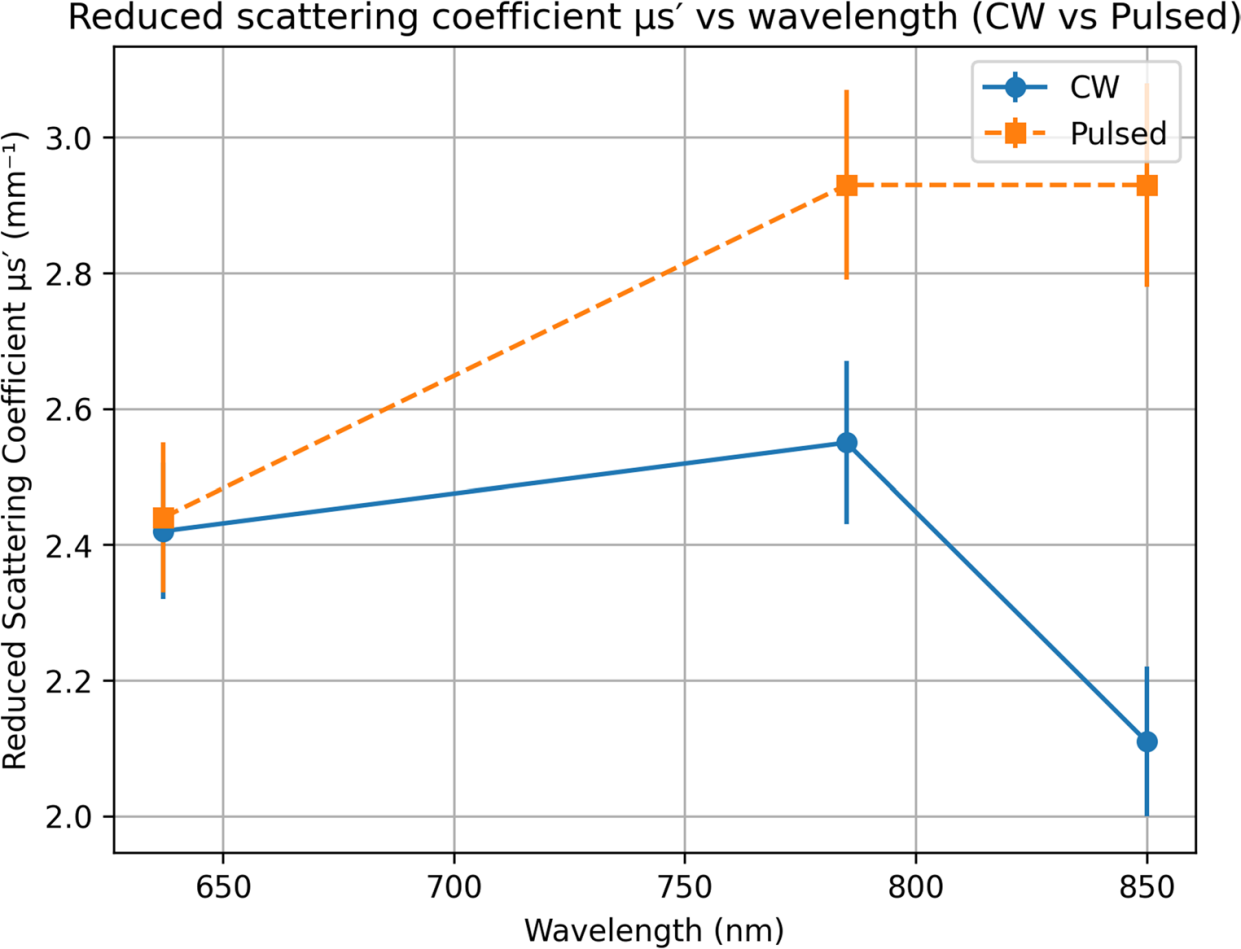
Reduced scattering coefficient (µs′) as a function of wavelength under continuous-wave (CW) and pulsed laser irradiation. Data are presented as mean ± standard deviation (SD) (*n* = 20 measurements distributed across three independent samples). Error bars represent SD

As shown in Fig. 5, although the increase in µs′ under pulsed illumination does not represent a strict two-fold change, the relative enhancement (~ 35–40%) is substantial in the context of biological tissue optics and exceeds the variability observed at shorter wavelengths.

### Summary of statistical findings

To improve clarity and consistency, statistical results have been integrated into the corresponding subsections of the Results section (Sect. 3.1–3.7), where relevant p-values are presented together with each optical parameter. This subsection provides a concise summary of the overall statistical trends obtained from the two-way ANOVA analysis.

The statistical evaluation demonstrated that wavelength exerted a significant effect on the scattering coefficient (µs) and anisotropy factor (g) (*p* < 0.001), indicating that photon scattering behavior in RCC tissue is strongly wavelength-dependent. In contrast, the absorption coefficient (µa) did not show statistically significant variation with either wavelength or laser irradiation mode (*p* > 0.05), despite exhibiting a monotonic spectral decrease.

Laser irradiation mode (continuous-wave versus pulsed) did not have a statistically significant effect on µs or g (*p* > 0.05), suggesting that the overall scattering magnitude and directionality are primarily governed by intrinsic tissue structure rather than by temporal modulation. However, the reduced scattering coefficient (µs′) exhibited a statistically significant dependence on laser irradiation mode, particularly at 850 nm, where a pronounced increase was observed under pulsed illumination (*p* = 0.0286).

Furthermore, a significant wavelength × laser mode interaction effect was identified for µs′ (*p* < 0.001), indicating that the influence of pulsed irradiation on scattering behavior is strongly wavelength-dependent. This interaction highlights the sensitivity of µs′ to modulation-induced microstructural or refractive index variations within RCC tissue.

Overall, these findings confirm that while absorption and total scattering remain relatively stable under different irradiation modes, the reduced scattering coefficient (µs′) is the most responsive parameter to laser modulation, particularly in the near-infrared spectral region.

## Discussion

This study presents a comprehensive optical characterization of renal cell carcinoma (RCC) tissue across multiple wavelengths and laser irradiation modes, providing new insights into wavelength- and modulation-dependent light–tissue interactions in malignant renal tissue. By combining multi-wavelength diode laser measurements with continuous-wave and pulsed delivery modes and rigorous IAD-based reconstruction, several important observations emerge that are directly relevant to optical diagnostics and laser-based therapeutic applications.

### Wavelength effects and biological interpretation

Wavelength exerted a statistically significant influence on the scattering coefficient (µs), anisotropy factor (g), and reduced scattering coefficient (µs′), while the absorption coefficient (µa) exhibited a monotonic spectral trend without reaching statistical significance. Absorption decreased from 637 nm to 850 nm, reflecting reduced hemoglobin absorption in the near-infrared region. In contrast, scattering magnitude and directionality varied with wavelength, indicating that photons at longer wavelengths experience fewer directional changes and achieve deeper penetration within RCC tissue.

The observed wavelength-dependent scattering behavior is also consistent with fundamental light–tissue interaction theory. The scattering coefficient exhibited a wavelength-dependent but non-monotonic behavior, with elevated values at 785 nm and lower values toward the near-infrared region. This trend can be explained by the reduced refractive index mismatch at near-infrared wavelengths and the increased contribution of larger intracellular scatterers, such as nuclei, mitochondria, and lipid-rich inclusions. As a result, photons at 850 nm undergo fewer angular deviations and achieve greater penetration depths within tumor tissue. From a therapeutic perspective, this behavior is advantageous for applications such as laser ablation, interstitial photothermal therapy, and deep-seated photodynamic therapy, where enhanced penetration and controlled energy deposition are critical.

### Comparison with healthy kidney tissue

When compared with previously reported optical properties of healthy kidney tissue in the near-infrared region—characterized by higher scattering coefficients (µs ≈ 10–15 mm⁻¹) and stronger forward scattering (g ≈ 0.75–0.85)—RCC tissue in the present study exhibited substantially reduced scattering and a more isotropic angular distribution. Baran et al., [2] demonstrated the feasibility of optical property measurements for RCC in photodynamic therapy planning. More recently, Botelho et al., [5] provided spectral optical comparisons between healthy and pathological kidney tissues. This deviation underscores the profound impact of tumor-driven microarchitectural remodeling on optical behavior.

Histopathological hallmarks of RCC, including loss of normal tubular organization, accumulation of extracellular fluid, nuclear pleomorphism, enlargement of intracellular organelles, and the presence of lipid-rich clear-cell cytoplasm, collectively disrupt well-defined refractive index boundaries within the tissue. Baldewijns et al., [1] reported increased angiogenic activity in high-grade RCC. Li et al., [12] further showed that microstructural alterations significantly influence optical properties in malignant tissues. These structural alterations increase spatial disorder and reduce coherent forward scattering, leading to lower g values and diminished µs relative to healthy renal parenchyma. Such changes are consistent with established optical models linking tissue ultrastructure to scattering anisotropy and provide a mechanistic basis for distinguishing malignant from healthy kidney tissue using optical methods. Jacques [11], established the theoretical framework for light–tissue interactions. Tuchin [18], expanded these principles and provided detailed models for scattering in biological tissues.

**Fig. 6:**
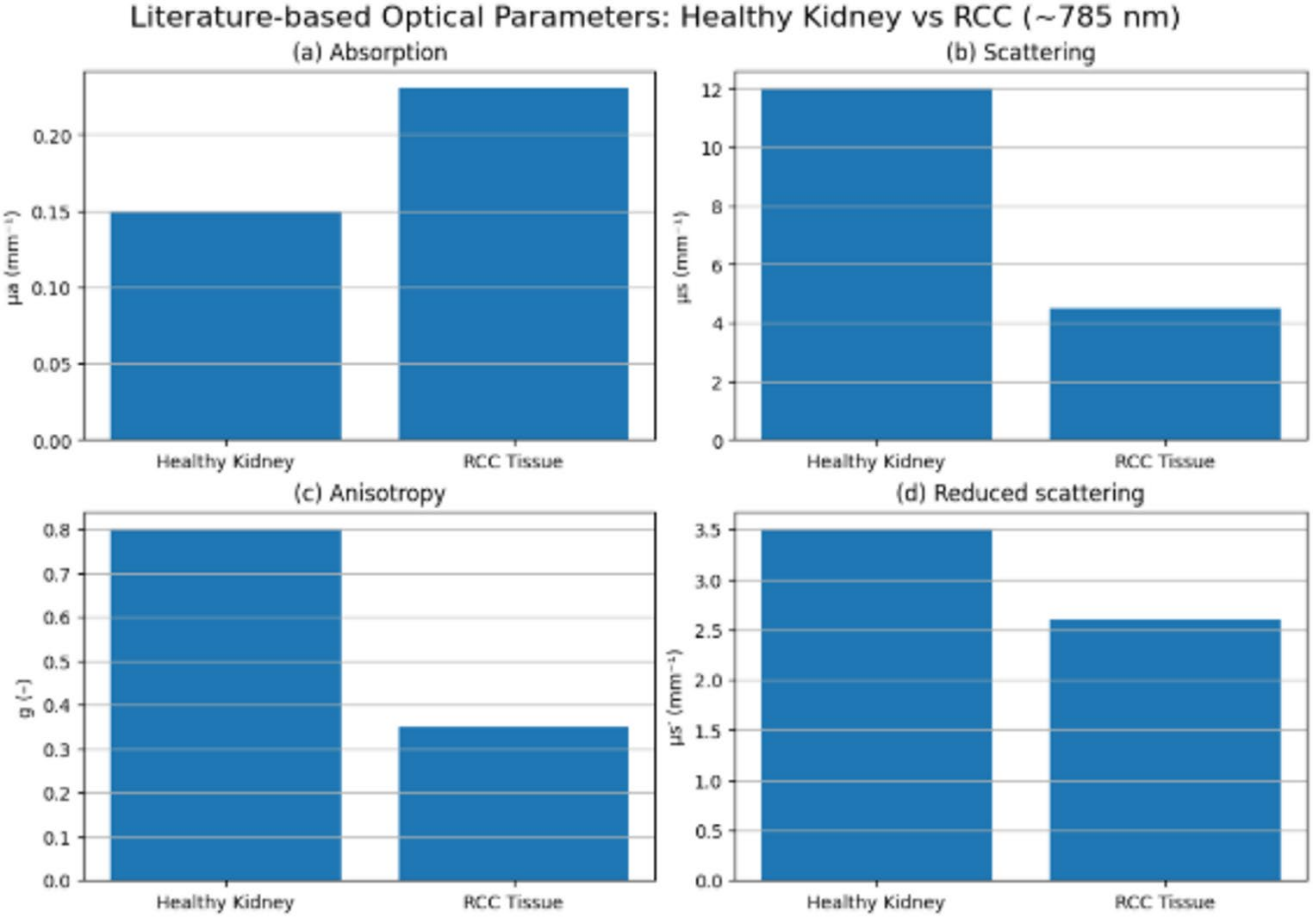
Literature-based comparison of optical parameters of healthy kidney and renal cell carcinoma (RCC) tissue in the near-infrared region (~ 785 nm): **a** absorption coefficient (µa), **b** scattering coefficient (µs), **c** anisotropy factor (g), and **d** reduced scattering coefficient (µs′). Representative values were selected from diffuse reflectance–based optical studies to illustrate characteristic differences between healthy and malignant renal tissues

As shown in Figs. 6b and c, tumor-associated structural alterations manifest optically as substantially lower scattering coefficients and anisotropy factors in RCC tissue (µs ≈ 3–5 mm⁻¹, g ≈ 0.30–0.40) compared with healthy renal parenchyma. These findings are consistent with the breakdown of normal tubular organization, increased extracellular fluid content, and intracellular heterogeneity characteristic of malignant transformation. Despite these pronounced differences in µs and g, the reduced scattering coefficient µs′ (Fig. 6d) remains of comparable magnitude for healthy and malignant tissues (≈ 2.5–3.5 mm⁻¹). This apparent convergence arises from the relationship µs′ = µs(1 − g), whereby reduced forward directionality partially compensates for the lower scattering amplitude, resulting in similar effective photon diffusion behavior at tissue-relevant length scales.

In addition to scattering-related parameters, absorption exhibits systematic differences between healthy kidney and RCC tissue (Fig. 6a). At approximately 785 nm, RCC tissue shows moderately higher µa values (≈ 0.20–0.25 mm⁻¹) than healthy parenchyma (≈ 0.10–0.18 mm⁻¹), consistent with increased vascular density and hemoglobin content associated with tumor angiogenesis. When µs′ values are comparable, this absorption contrast becomes a dominant factor governing optical penetration depth and spatial energy deposition under diagnostic and therapeutic illumination conditions. Collectively, these observations underscore that µs′—rather than µs or g alone—provides a more physiologically meaningful descriptor of photon propagation in structurally heterogeneous renal tumors, highlighting the importance of complete optical parameterization for laser-based diagnostics and therapies in RCC.

### Modulation effects and novel µs′ behavior

One of the most notable findings of this study is the statistically significant increase in µs′ observed under pulsed illumination at 850 nm. To the best of our knowledge, such a pronounced modulation-dependent enhancement of µs′ has not been previously reported for renal tissue. Several complementary mechanisms may contribute to this behavior. First, even modest transient temperature rises (≤ 1 °C) during millisecond-scale laser pulses can induce microthermal expansion of intracellular scatterers, such as organelles, thereby increasing refractive index mismatch and effective scattering strength. Second, pulsed irradiation may give rise to reversible thermo-optic or thermoelastic refractive index modulations within cellular and subcellular components. Third, at 850 nm—where hemoglobin absorption is minimal—incident photons predominantly interact with scattering structures rather than chromophores, rendering scattering-related parameters particularly sensitive to transient structural perturbations.

The statistically significant increase in µs′ under pulsed 850 nm illumination suggests modulation-dependent microstructural dynamics that are not captured under continuous-wave delivery. Pulsed irradiation can induce rapid thermoelastic stress cycles, leading to nanometric-scale expansion and relaxation of cellular and extracellular structures. Such transient mechanical fluctuations can momentarily alter local refractive index distributions, increasing angular redistribution of scattered photons. In addition, subcellular organelles, particularly mitochondria and lipid droplets that are abundant in clear cell RCC, may undergo thermally driven swelling kinetics, producing short-lived changes in scattering cross-sections. Together, these effects provide a plausible explanation for why µs′—but not µs or µa—exhibits heightened sensitivity to pulsed illumination at near-infrared wavelengths. While the precise biophysical origin of this modulation-dependent behavior requires further time-resolved and temperature-resolved investigation, the present findings clearly demonstrate that the reduced scattering coefficient (µs′) is significantly influenced by the temporal characteristics of laser delivery and represents a highly sensitive parameter for probing dynamic light–tissue interactions in renal cell carcinoma. Additionally, the use of a relatively large beam diameter (5 mm) enables spatial averaging over heterogeneous tissue regions, thereby minimizing local anisotropic effects. While beam size may influence localized scattering behavior, it is not expected to significantly affect bulk optical parameter estimation under the present experimental conditions.

### Clinical and modeling implications

The findings of this study have several important implications for laser-based diagnosis and treatment of RCC. First, the observed increase in µs′ under pulsed illumination suggests enhanced angular randomization and potentially greater superficial energy confinement, which may be advantageous for optimizing photothermal therapy protocols. Second, the sensitivity of µs′ to both wavelength and laser modulation highlights its potential utility as an optical biomarker of RCC microstructural heterogeneity in diagnostic imaging applications. Third, these results indicate that light transport models—particularly Monte Carlo and diffusion-based simulations—should incorporate temporal laser modulation effects, which have largely been neglected in existing RCC optical models. Finally, for photodynamic therapy applications, modulation-dependent changes in µs′ imply that pulsed light delivery may alter intratumoral light distribution and treatment depth, underscoring the need for modulation-aware dosimetry in clinical practice.

### Clinical translation

The wavelength- and modulation-dependent optical behavior observed in this study has direct implications for the clinical translation of laser-based diagnostic and therapeutic techniques in renal cell carcinoma. As summarized in Table 4, continuous-wave (CW) and pulsed irradiation modes offer distinct advantages depending on the selected wavelength and intended clinical application. These differences arise primarily from the interplay between absorption, scattering magnitude, and scattering directionality, which together determine optical penetration depth, energy confinement, and spatial light distribution within tumor tissue.

At 637 nm, CW illumination provides stable absorption with limited penetration depth, making it well suited for surface-confined applications such as superficial photodynamic therapy (PDT) and surgical margin assessment. Under pulsed operation at the same wavelength, the slight increase in the reduced scattering coefficient (µs′) enhances angular photon redistribution, which may improve contrast in optical biopsy and superficial imaging modalities without substantially altering thermal load.

At 785 nm, CW illumination benefits from reduced absorption and moderate penetration depth, supporting applications such as Raman spectroscopy and deeper optical imaging. Pulsed delivery at this wavelength introduces modulation-sensitive changes in µs′, suggesting potential utility in microstructural contrast imaging, where sensitivity to subcellular organization and tumor heterogeneity is desirable.

At 850 nm, CW illumination achieves the greatest penetration depth with stable thermal diffusion, characteristics that are advantageous for photothermal therapy (PTT) and interstitial laser treatments. In contrast, pulsed illumination at 850 nm produces a pronounced increase in µs′, leading to enhanced angular scattering and redistribution of deposited energy. This behavior may be exploited to improve ablation precision, achieve selective heating of tumor regions, and reduce unintended thermal spread to surrounding healthy tissue.

Collectively, these findings demonstrate that optimal clinical implementation of laser-based technologies for RCC requires careful selection of both wavelength and temporal delivery mode. Incorporating modulation-dependent optical parameters—particularly µs′—into treatment planning and diagnostic protocols may improve therapeutic precision and diagnostic sensitivity, thereby facilitating more effective and personalized laser-based interventions.

**Table 4:** Clinical implications of continuous-wave and pulsed laser irradiation modes at different wavelengths for renal cell carcinoma applications

Wavelength	Mode	Clinical Advantage	Suitable Applications
637 nm	CW	Stable absorption, shallow penetration	Surface PDT, margin assessment
637 nm	Pulsed	Slightly increased µs′ → better contrast	Optical biopsy, superficial imaging
785 nm	CW	Good penetration, low absorption	Raman spectroscopy, deep imaging
785 nm	Pulsed	Modulation-sensitive µs′	Microstructural contrast imaging
850 nm	CW	Deepest penetration, stable thermal spread	PTT, interstitial laser therapy
850 nm	Pulsed	Significant µs′ increase → energy redistribution	Ablation precision, selective heating

### Limitations

This study has several limitations that should be acknowledged. First, the analysis was restricted to the clear cell subtype of renal cell carcinoma, and optical properties may differ in other histological variants such as chromophobe or papillary RCC. Second, although the number of measurements was sufficient to reveal statistically significant trends, the overall sample size was limited, and larger patient cohorts may better capture inter-patient variability and tumor heterogeneity. Third, all measurements were performed ex vivo; therefore, physiological factors present in vivo—including blood perfusion, oxygenation, and active thermoregulation—may alter tissue optical behavior. While temperature and hydration were carefully controlled during data acquisition, subtle deviations may still influence scattering-related dynamics. Finally, the integrating sphere–IAD methodology assumes optical homogeneity within each tissue section. Given the inherently heterogeneous microstructure of RCC, microscale spatial variations in optical properties may not be fully resolved using this bulk-averaged approach. Future studies will focus on in vivo measurements, larger patient cohorts, and time-resolved optical techniques to further investigate dynamic changes in scattering behavior under different laser modulation conditions. High-sensitivity optical approaches may also enable the identification of steady and evolving biological states in cancer tissues, as suggested in recent studies (Sensors, 2019, 19, 4728).

## Conclusion

This study provides a comprehensive multi-wavelength optical characterization of renal cell carcinoma (RCC) tissue, incorporating both continuous-wave and pulsed diode laser illumination at 637, 785, and 850 nm. By employing the Inverse Adding–Doubling (IAD) method, absorption, scattering, anisotropy, and reduced scattering coefficients were reconstructed with high numerical accuracy, enabling systematic evaluation of wavelength- and modulation-dependent optical behavior with clear biological relevance.

Across all investigated wavelengths, RCC tissue exhibited markedly lower scattering coefficients and reduced anisotropy compared with reported values for healthy kidney tissue. These differences are consistent with the disrupted microarchitecture of malignant renal tissue, including loss of normal tubular organization, increased extracellular fluid content, nuclear pleomorphism, and lipid-rich clear cell morphology. Such structural alterations result in a more isotropic optical response and increased variability in the reduced scattering coefficient (µs′), reflecting the intrinsic heterogeneity of RCC.

Wavelength significantly influenced the scattering-related optical parameters (µs, g, and µs′), whereas absorption (µa) remained statistically insensitive to wavelength and laser modulation. In contrast, reduced scattering (µs′) demonstrated a pronounced dependence on laser irradiation mode, particularly under pulsed illumination at 850 nm, emphasizing the importance of temporal laser parameters in modeling and optimizing light–tissue interactions in renal cell carcinoma.

Laser modulation further influenced RCC optical behavior. While absorption and total scattering showed no significant dependence on continuous-wave versus pulsed illumination, the reduced scattering coefficient (µs′) exhibited statistically significant modulation-dependent variations. These changes suggest the presence of transient microstructural or refractive index dynamics that are not captured by conventional steady-state optical models. Importantly, direct temperature measurements confirmed that laser irradiation did not produce a measurable macroscopic temperature rise during the experiments, indicating that the observed effects are not attributable to bulk thermal heating but rather to subtle, transient optical or thermoelastic processes.

Overall, these findings emphasize the importance of precise optical property characterization for optimizing laser–tissue interaction models, improving phototherapy dosimetry, and advancing minimally invasive optical diagnostics for renal malignancies. This work establishes a foundational dataset for RCC optical behavior and underscores the necessity of incorporating both wavelength and temporal modulation parameters into next-generation laser-based treatment planning and imaging strategies.

## Data Availability

The data generated and analyzed during this study are available from the corresponding author upon reasonable request.
